# World TB Day — March 24, 2013

**Published:** 2013-03-22

Each year, World TB Day is observed on March 24. This annual event commemorates the date in 1882 when German bacteriologist Robert Koch announced his discovery of *Mycobacterium tuberculosis*, the bacillus that causes tuberculosis (TB). World TB Day provides an opportunity to raise awareness about TB-related problems and solutions, and to support worldwide TB control efforts. For the second year, CDC joins the global Stop TB Partnership in adopting the World TB Day slogan, “Stop TB in My Lifetime.”

In 2012, a total of 9,951 new TB cases were reported in the United States, for a rate of 3.2 cases per 100,000 (1). This is the first time the number of TB cases has dropped below 10,000 since standardized national reporting began in 1953. Despite this milestone, a number of challenges remain that slow progress toward the goal of TB elimination in the United States. TB still persists in specific populations; foreign-born persons, racial/ethnic minorities, and homeless persons continue to be affected disproportionately (2).

CDC is committed to a world free of TB. Initiatives to improve awareness, testing, and treatment of latent TB infection and TB disease among high-risk groups are critical to reach the goal of TB elimination in the United States. Additional information about World TB Day and CDC’s TB elimination activities is available at http://www.cdc.gov/tb/events/worldtbday.
